# Modelling and performance analysis of clinical pathways using the stochastic process algebra PEPA

**DOI:** 10.1186/1471-2105-13-S14-S4

**Published:** 2012-09-07

**Authors:** Xian Yang, Rui Han, Yike Guo, Jeremy Bradley, Benita Cox, Robert Dickinson, Richard Kitney

**Affiliations:** 1 Department of Computing, Imperial College London, London, SW7 2AZ, UK; 2 Business School, Imperial College London, London, SW7 2AZ, UK; 3 Department of Bioengineering, Imperial College London, London, SW7 2AZ, UK

## Abstract

**Background:**

Hospitals nowadays have to serve numerous patients with limited medical staff and equipment while maintaining healthcare quality. Clinical pathway informatics is regarded as an efficient way to solve a series of hospital challenges. To date, conventional research lacks a mathematical model to describe clinical pathways. Existing vague descriptions cannot fully capture the complexities accurately in clinical pathways and hinders the effective management and further optimization of clinical pathways.

**Method:**

Given this motivation, this paper presents a clinical pathway management platform, the Imperial Clinical Pathway Analyzer (ICPA). By extending the stochastic model performance evaluation process algebra (PEPA), ICPA introduces a clinical-pathway-specific model: clinical pathway PEPA (CPP). ICPA can simulate stochastic behaviours of a clinical pathway by extracting information from public clinical databases and other related documents using CPP. Thus, the performance of this clinical pathway, including its throughput, resource utilisation and passage time can be quantitatively analysed.

**Results:**

A typical clinical pathway on stroke extracted from a UK hospital is used to illustrate the effectiveness of ICPA. Three application scenarios are tested using ICPA: 1) redundant resources are identified and removed, thus the number of patients being served is maintained with less cost; 2) the patient passage time is estimated, providing the likelihood that patients can leave hospital within a specific period; 3) the maximum number of input patients are found, helping hospitals to decide whether they can serve more patients with the existing resource allocation.

**Conclusions:**

ICPA is an effective platform for clinical pathway management: 1) ICPA can describe a variety of components (state, activity, resource and constraints) in a clinical pathway, thus facilitating the proper understanding of complexities involved in it; 2) ICPA supports the performance analysis of clinical pathway, thereby assisting hospitals to effectively manage time and resources in clinical pathway.

## Introduction

Today, hospitals are asked to serve more and more patients while maintaining the quality of healthcare with limited medical staff and equipment. This situation causes many serious problems, including overcrowded emergency departments, delayed treatment of urgent patients, long waiting time and decreasing satisfaction of both doctors and patients [1]. Within such a context, it becomes essential to apply information and communications technology (ICT) to achieve more efficient hospital management. Health informatics (also named clinical informatics) applies ICT to healthcare and biomedicine for promoting public health, facilitating hospital management and reducing healthcare cost [2]. Among its various branches, clinical pathway, emerging in the 1980s [3], is a popular tool to outline the sequence and timing of actions necessary to a desired outcome with optimal efficiency [4]. The sequence of clinical actions performed by a multidisciplinary team moves a patient with a specific diagnosis progressively through a clinical experience to a desired healthcare effect [4,5].

Clinical pathway focuses on improving the efficiency of the healthcare process for patients, especially those with acute diseases. A number of factors affect clinical pathways, including changing amount of incoming patients, uncertain diagnosis duration and other unpredictable events. To date, the lack of formal modelling methods in existing techniques [6-13] means the description of clinical pathway is rather vague which results that the complex behaviours involved in clinical pathway cannot be adequately understood by hospital managers. For such an issue, a mathematical model is required to accurately describe these behaviours of a clinical pathway. A novel platform for more efficient clinical pathway management, therefore, is proposed in this paper. The paper features four key elements:

1. A clinical pathway management platform, Imperial Clinical Pathway Analyser (ICPA), is introduced for quantitatively analysing clinical pathway. This platform can construct models of clinical pathway using existing clinical data and conducts performance analysis based on these models. The analysis results can benefit hospitals by providing crucial information for clinical pathway management.

2. Developed from performance evaluation process algebra (PEPA) [14], a stochastic model clinical pathway PEPA (CPP) is introduced to accurately describe different aspects of a clinical pathway, including its state transitions and treatment activities as well as their associated resources and constraints.

3. Using CPP, performance analysis of a clinical pathway can be conducted. The analysis results provide a variety of useful information in the clinical pathway. Firstly, resource utilisation can help hospitals to optimise resource allocation. Secondly, the passage time under different patient inputs shows patients' expected residing time in hospitals. Thirdly, the maximum number of input patients reveals the capacity of hospitals.

4. A stroke clinical pathway obtained from Charing Cross Hospital of Imperial College London is demonstrated. We choose this example for medical and economic reasons. Firstly, stroke is a typical acute disease and the third most common cause of death worldwide. Hence any delay in treatment may result in severe disability [15,16]. Secondly, according to a recent National Audit Office report, 4-6% of the total NHS expenditure in the UK is spent on stroke treatment. It is predicted that the better management of stroke care can bring £20 m in annual savings, 550 fewer deaths and 1700 fewer cases of disability in the UK [17,18].

### Motivation

**Figure 1:**
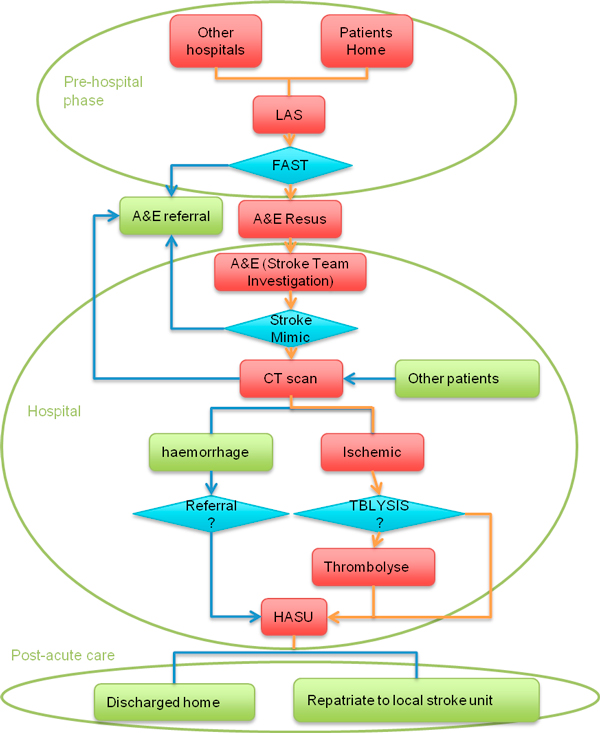
**An example of the stroke clinical pathway**.

The stroke clinical pathway investigated in this paper is for illustrative purposes. Stroke is a complex disease requiring a systematic integration of services, e.g. primary care, ambulance services, acute treatment and rehabilitation, post-acute rehabilitation, and often long-term health and care support in the community. Due to its complexity and requirement of various services, the stroke clinical pathway presents a significant challenge to existing generally fragmented health and social care services. Moving from the current fragmented approach to an integrated stroke system is a complex task. As simulation models are useful during the planning stage of complex service re-configuration, modelling methods are in demand to simulate and optimize the stroke clinical pathway. Figure 1 displays the abstraction of a general stroke clinical pathway, obtained from Charing Cross hospital of Imperial College London. A new 999 call from a patient or other hospitals initiates an instance of this pathway. The information flow is summarized as follows:

1. First, an ambulance scheduled by London Ambulance Service (LAS) picks up the patient, where a Fitness and Anthropometric Scoring Template (FAST) test is carried out.

2. The patient is sent to the accident and emergency (A&E) resuscitation department and referred to the stroke team, if the FAST test shows s/he potentially has a stroke. Otherwise s/he is sent to the normal emergency department.

3. Next, if the patient is further diagnosed to have a stroke, s/he is sent to the CT department; otherwise s/he is transferred to the normal A&E department.

4. If a CT scan shows the patient does not have a stroke, s/he will be switched to the non-stroke treatment department. If the patient has a haemorrhagic stroke, s/he is transferred to the Hyper-Acute Stroke Unit (HASU). If the patient has an ischemic stroke, clinicians need to further determine whether the patient needs thrombolysis therapy.

5. Afterwards, the patient is sent to the HASU to be cared by occupational therapists for two or three days.

6. Finally, the patient is discharged from hospital, and the community therapy team takes over.

As every patient has his/her own journey, modulated by clinical issues and availability of resources, the clinical pathway in Figure 1 is a model of a highly dynamic system. To model such a system, two challenges need to be addressed:

1. There are many uncertainties in the clinical pathway, so that a model may need to simulate pathways in a stochastic manner. For example, the stroke clinical pathway in Figure 1 has a changing number of incoming patients, and the time spent in each department for different patients may vary significantly. Patients with different diagnoses will be transferred to different departments, taking different routes through the pathway. Therefore a modelling method, which can cope with these complexities, is required.

2. Performance analysis for different scenarios should be supported. For example, patients would be interested in the expected time they would spend in hospital, while hospitals need to estimate the maximum number of patients they can treat every day and manage the clinical pathway in a cost-effective way. Thus a clinical pathway management platform, which can explicitly evaluate the clinical pathway performance, such as passage time and throughput, would be of interest.

### Related work

The general impact of information technology on the clinical pathway like the influence of IT support on the satisfaction of both patients and medical staff is studied [8-10]. The clinical pathway can be implemented manually on paper [11] or electronically [12]. With a good understanding of the clinical pathway, a set of evidence-based recommendations forming guidelines for clinical practice can be developed to both formalize and optimize the care process [13]. Thus IT techniques embedded into clinical pathways can efficiently decrease undesired practice variability and improve clinician performance.

Some researchers further propose methods of modelling the clinical pathway. By using quantitative models, the influence of individual resources on overall pathway performance can be evaluated directly. However, little work has been done in the area of modelling clinical pathways with the aim of quantitatively improving system performance. Although in [19], a pathway model to facilitate the stroke care planning process is introduced, without a mathematical representation, the model performance cannot be shown explicitly. The work has been done in [20] applies the workflow graph to model the clinical pathway. Using this model, a similarity function is proposed to evaluate the temporal equivalence of two clinical pathways in order to reduce a complex pathway scenario into a simple one. In [21], ontology is used to describe the clinical pathway. This model allows the hierarchical representation of the clinical pathway, e.g., a clinical pathway can be described as a combination of a high-level outcome flow and a detailed workflow with care time constraints. However, the work done in [20] and [21] cannot build a model to explicitly measure the performance of the clinical pathway, such as throughput and passage time. Some researchers focus on analysing time and resource information in clinical pathway using stochastic Petri net. In [22], Rui et al. introduce the Probabilistic Time Constraint WorkFlow Nets (PTCWF-nets) to model a process. A static analysis method is then proposed to analyse each activity's probability of meeting its time constraints (e.g., deadline) in a process before the process is actually executed. In addition, they develop a dynamic method during the execution of the process [23]. This method can update remaining activities' probabilities of successful execution whenever some activities are completed and their actual durations are known. Time schedulability is only one aspect of performance analysis in clinical pathway and it is closely related to another aspect: resource analysis. In [24], they further apply PTCWF-nets to manage resources. Their approach can schedule a clinical pathway among multiple available resources and allocate each activity to an optimal resource, i.e., the resource that has the highest probability to finish the activity in time. Authors in [25] also attempt to model clinical pathway using Petri net. They introduce performance trees to provide a standard unifying framework for expressing performance measures and performance requirements. Benefit from performance trees, the Petri net model for clinical pathway can provide estimation of steady state distribution and passage time.

Besides the work has been done to model the clinical pathway using Petri net, process algebra is also introduced to formally specify the interactions of different entities in the clinical pathway [26]. Process algebra is a mathematical framework used to describe a complex parallel system. In this framework, both the behaviour and properties of the system are described in the form of algebra, facilitating accurate definition and rigorous reasoning about the system in mathematics. PEPA is an enhanced process algebra mainly used to describe and analyse the performance of concurrent systems [14]. It inherits most characteristics of process calculus while incorporating features to specify a stochastic model, which potentially behaves as a continuous time Markov process. Comparing PEPA with other frequently used modelling tools, a queuing network offers compositionality but lacks formal definition, while a Petri net has formal definition without good compositionality. Thus in paper [27], PEPA is used to model the healthcare system. Based on PEPA, the execution duration and throughput of the clinical pathway can be evaluated. However, the role of resources, which constrain the activities and further limit pathway outcome, is not explicitly shown in this model. Therefore, this paper proposes a general clinical pathway model based on the CPP to analyse the performance of system and optimize pathway output.

The remainder of this paper is organised as follows: the *Method *Section introduces the architecture of ICPA, defines the CPP model, and presents key theories in conducting performance analysis on clinical pathway; the *Results *Section reports our experimental tests of the CPP model's effectiveness; finally, the *Conclusion *Section summarises our work.

## Method

**Figure 2:**
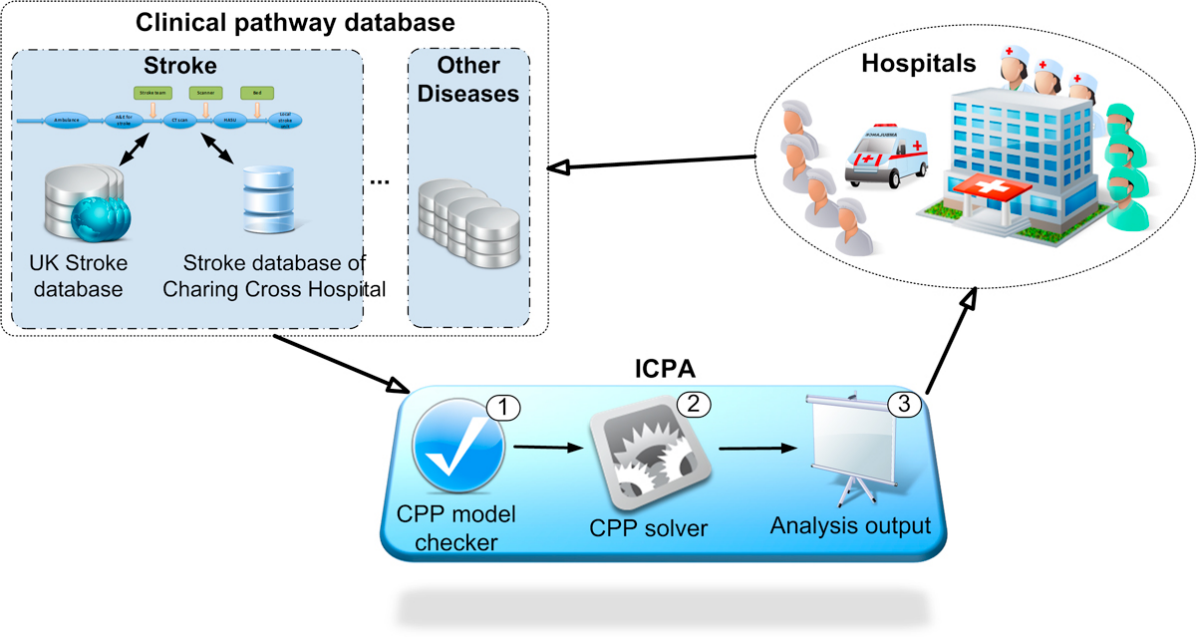
**The architecture of ICPA platform**.

A novel clinical pathway management platform, ICPA, is proposed to effectively manage clinical data and provide feedback to hospitals, aiming to improve healthcare systems by constructing models of clinical pathways. The architecture of ICPA is shown in Figure 2. Hospitals, treating various patients, take records of patients' treatment processes. The collected clinical data is stored in clinical pathway databases. As the specific disease studied in this paper is stroke, ICPA uses national stroke related databases, such as the Stroke Improvement National Audit Programme (SINAP) database [28], and the database containing stroke clinical data in Charing Cross hospital to parameterize the stroke clinical pathway model. By constructing the stroke clinical pathway model using our formal modelling method CPP, ICPA can analyse the performance of the clinical pathway, where throughput and passage time are estimated. Using analysis results, hospitals can efficiently reconfigure the clinical pathway. Hence, ICPA is a useful clinical pathway platform for hospitals to understand problems and find bottlenecks in the disease treatment process.

The core element of ICPA is the CPP modelling method. In ICPA the constructed model, whose modelling language should follow the rules of PEPA, is first checked by the CPP Model Checker. By applying the CPP modelling method, the clinical pathway can be modelled in a stochastic manner where the time duration of each activity in the disease treatment process is a variable. Furthermore, the CPP method supports multiple parallel patients, enabling competition among patients for the same treatment resources. As the underlying stochastic model of CPP is a continuous time Markov Chain, the steady state distribution and an estimation of passage time can be produced. In the following subsections, technical details of the CPP modelling method will be discussed.

### Definition of CPP

Inheriting and developed from PEPA, the basic elements of CPP are state components, resource components and activities. A state component represents the status of a patient by showing in which department the patient is being treated. A resource component is used to specify the state of each resource, which can be either busy or idle. The third component is the activity, which decides transitions between different state components. As each activity relies on one or more resources, the state transition can only take place when the associated resource components are in their idle states. Therefore, the number of resources constrains the frequency of state transitions and limits the system throughput. The model defined by CPP which represents states and resources separately is suitable for resource optimization and status monitoring of patients. Definitions of CPP are as follows.

**Definition 1**. *In CPP, the clinical pathway is represented by a five-tuple, < S*, *R*, *Act*, *C*, *F_C _>, where:*

• *S is a finite set of states s *∈ *S, showing places that patients are being treated;*

• *R is a finite set of resources, r *∈ *R, required during treatment;*

• *Act is a finite set of activities a *∈ *Act; Each activity, a, is represented by a two-tuple *(*α*, *rate*), *where α is the action type and rate is one over the mean value of execution duration which is an exponentially distributed random variable;*

• *C is a set of constraints c *∈ *C;*

• *F_C _is a set of functions that determine action rate: rate *= *f *(*c*_*r*1_, *c*_*r*2_, . . . , *c*_*rn*_) *where f *∈ *F_C_, c_ri _*∈ *C and *1 ≤ *i *≤ *n*.

**Definition 2**. *In CPP, the set S and R are basic components. Given that these components can be commonly denoted as P or Q, the syntax of the terms in CPP can be defined as follows:*(1)

• *The sequential operator '*.*' defines the order of P and Q;*

• *The choice operator '*+*' indicates competition between P and Q;*

• *The cooperation operator **determines the interaction between P and Q over the action set L*.

For example, $P\overset{def}{=}\left(\alpha ,rate\right).Q$ means that the component P becomes *Q *with the completion of the activity (*α*, *rate*). The expression *P *+ *Q *represents that the system can behave either as *P *or *Q*. It enables all the activities of *P *and *Q*, and the first completed activity determines how the system behaves. The cooperation operator in the expression  forms the basis of composition and can specify two components working cooperatively with shared activities defined in *L*.

### Three key parts of the CPP model

The CPP model consists of three key parts, namely state definition, resource specification and system description, which are used to show how the number of resources in the healthcare system affects the total throughput and the overall response-time for treatment.

1. State definition

The state definition part shows how patients proceed through healthcare system. It consists of multiple state components representing places that patients are being treated. Based on their corresponding pathology states, patients with different diagnosis results are transferred to various departments. Two related state components are connected by sequential and choice operators. The sequential operator shows the time sequence of states, while the choice operator represents competition between two states. Here is an example of the state component definition:

(2) $$Patient_{place1}\;\overset{def}{=}\;\left(\alpha ,\rho_{place2}*rate_{\alpha }\right).Patient_{place2}+\left(\alpha ,\rho_{place3}*rate_{\alpha }\right).Patient_{place3}$$

It shows that patient can move from the state *Patient*_*place*1 _to *Patient*_*place*2 _or *Patient*_*place*3 _with probabilities of *ρ*_*place*2 _and *ρ*_*place*3 _after the completion of the activity whose action type is *α*.

2. Resource specification

The resource specification part defines all resources including equipment and medical staff required by state components. Transition between different state components requires the availability of corresponding resources. Assume the resource *Resource*_*place*1 _needed by the state component *Patient*_*place*1 _is defined as

(3) $$\begin{array}{ccc} Resource\text{\_}idle_{place1}\; & \overset{def}{=}\;\left(\beta ,rate_{\beta }\right).Resource_{-}busy_{place1} & \\ Resource\text{\_}busy_{place1}\; & \overset{def}{=}\;\left(\alpha ,rate_{\alpha }\right).Resource_{-}idle_{place1} \end{array}$$

where *Resource_busy*_*place*1 _represents that the resource is currently busy in completing the activity whose action type is *α*, while *Resource*_*idle*_*place*1 _shows the resource is currently idle waiting for the completion of activity whose action type is *β*. The activities with action type of *β *and *α *can occur in:

(4) $$\begin{array}{ccc} Patient_{place0}\; & \overset{def}{=}\;\left(\beta ,rate_{\beta }\right).Patient_{place1} & \\ Patient_{place1}\; & \overset{def}{=}\;\left(\alpha ,rate_{\alpha }\right).Patient_{place2} \end{array}$$

where the state *Patient*_*place*0 _is the previous state of *Patient*_*place*1 _and *Patient*_*place*2 _is the next state of *Patient*_*place*1_. With completion of the activity whose action type is *β*, patients can move from *Patient*_*place*0 _to *Patient*_*place*1_. Meanwhile, the resource required by *Patient*_*place*1 _becomes busy in treating the patient. After the treatment, denoted as the activity with the action type of *α*, the resource becomes idle again waiting to treat another patient.

3. System description

The last part of the CPP model is the system description part, describing the whole clinical pathway by using the cooperation operator to denote interactions between components. Suppose only one patient defined in Eqn.4 and one resource component defined in Eqn.3 are involved in the CPP model. The cooperation between state component *Patient*_*place*0 _and the resource component *Resource_idle*_*place*1 _can be represented as(5)

where *β *and *α *are the action types on which two components *Patient*_*place*0 _and *Resource*_ *idle*_*place*1 _synchronise. More specifically, the patient which is currently in the initial state *Patient*_*palce*0 _can only be scheduled to the following states when successive treatments are carried out.

As the healthcare system usually contains multiple patients and multiple copies of resources, it is necessary to represent parallel patients and resources as follows:

(6) $$\begin{array}{ccc} Patient_{place0}\left[Num_{Patient}\right]\; & \overset{def}{=}\;\underset{Num_{Patient}}{\underset{︸}{Patient_{place0}||...||Patient_{place0}}} & \\ Resource_{-}idle_{place1}\left[Num_{Re\text{s}ource1}\right]\; & \overset{def}{=}\;\underset{Num_{Resource1}}{\underset{︸}{Resource\text{\_}idle_{place1}||...||Resource\text{\_}idle_{place1}}} \end{array}$$

where || is the parallel combinator, equivalent to , showing that two components are in parallel. Therefore, we can describe the system which consists of multiple *Patient*_*place*0 _and *Resource*_*idle*_place1 _in the form of(7)

Although definitions in Eqn.3,4,7 can emphasize the influence of resource on patient health care process, it has a limitation. When all copies of the resource are in their busy state *Resource*_*busy*_*place*1_, a new coming patient cannot be transferred from *Patient*_*place*0 _to *Patient*_*place*1_. The patient will be stuck in the state *Patient*_*place*0 _until at least one copy of resource become idle enabling it transfer to *Patient*_*place*1 _and then to *Patient*_*place*2_. In reality, even if all the copies of resource are busy, we still want the new coming patient to be able to move from *Patient*_*place*0 _to *Patient*_*place*1 _and then stay in *Patient*_*place*1 _waiting for at least one copy of the resource becoming idle. Therefore, we need to revise the resource definition part by inserting an additional resource as follows:

(8) $$\begin{array}{ccc} Wait\text{\_}room0\text{\_}idle\; & \overset{def}{=}\;\left(\beta ,rate_{\beta }\right).Wait\text{\_}room0\text{\_}busy & \\ Wait\text{\_}room0\text{\_}busy\; & \overset{def}{=}\;\left(\gamma ,rate_{\gamma }\right).Wait\text{\_}room0\text{\_}idle\\ Resource\text{\_}idle_{place1}\; & \overset{def}{=}\;\left(\gamma ,rate_{\gamma }\right).Resource\text{\_}busy_{place1}\\ Resource\text{\_}busy_{place1}\; & \overset{def}{=}\;\left(\alpha ,rate_{\alpha }\right).Resource\text{\_}idle_{place1} \end{array}$$

Then the system description part is:(9)

The number of copies of the inserted resource *Wait_room*0_*idle *should be sufficiently large to guarantee that at least one copy is available at any time, that is *Num*_*Wait_room*0 _≥ *Num_Patient_*. Moreover, the action rate *rate*_*γ *_must be large enough to enable instant transition from *Wait*_*room*0_*busy *to *Wait*_*room*0_*idle *whenever at least one copy of resource is in its idle state *Resource*_*idle*_*place*1_.

With definitions in Eqn.4,8,9, the CPP model for a simple clinical pathway can be constructed. For more complex clinical pathway, we can augment this model to contain more state components and more resources.

### Key techniques of performance analysis using CPP

Rooted in a continuous time Markov process [14], CPP model can be used to estimate the performance of clinical pathway including *throughput *(the number of patients that the healthcare system can serve every day) and *resource utilisation *(the percentage of time that a resource is in use).

#### The underlying stochastic process

The basic idea of a Markov process is that the distribution of time until the next state change is independent of the time which has elapsed since the last state change. Let the state transition rate between states *S_i _*and *S_j _*be

(10) qSi,Sj= ∑α∈Si α→Sjrateα

If there are no direct connections between these two states, then the transition rate $q_{S_{i},S_{j}}=0$. Let *X*(*t*) = *S_i _*indicate that at time instance *t*, the system behaves as *S_i_*. After a tiny amount of time *δt*, the probability that the system is in state *S_j _*is

(11) $$Pr\left(X\left(t+\delta t\right)=Sj|X\left(t\right)=S_{i}\right)=q_{S_{i},Sj}*\delta t+O\left(\delta t\right),i\neq j$$

where *O*(*δt*) goes to zero faster than *δt*. Suppose that the infinitesimal generator for the process *Q *is a square matrix whose off-diagonal elements are $q_{S_{i},S_{j}}$ and diagonal elements are formed as the negative sum of the non-diagonal elements of each row, $q_{S_{i},S_{i}}=-\underset{j\neq i}{\sum }q_{S_{i},S_{j}}$. The evolution of the continuous time Markov process is represented by a first order differential equation [29]:

(12) P′(t)=Q*P(t)=P(t)*Q,where P(0)=I

where *P*(*t*) is a square matrix with (*i*, *j*)*th *entry *p*_*i*, *j *_standing for the probability *Pr*(*X*(*t*) = *S_j_*|*X*(0) = *S_i_*). As performance analysis is usually concerned with system behaviour over a significant period of time, it is necessary to study the steady state behaviour of the system. If the steady state distribution exists, then the proportion of time that the process spends in state *S_j _*is represented as:

(13) $$\pi_{j}=\underset{t\to ⊤}{\text{lim}}Pr\left(X\left(t\right)=S_{j}|X\left(0\right)=S_{0}\right)$$

Therefore, we can define:

(14) $$\prod =\underset{t\to ⊤}{\text{lim}}P\left(t\right)=\left[\begin{array}{cccc} \pi_{1} & \pi_{2} & \cdots \; & \pi_{N}\\ \pi_{1} & \pi_{2} & \cdots \; & \pi_{N}\\ \vdots & \vdots & & \vdots \\ \pi_{1} & \pi_{2} & \cdots \; & \pi_{N} \end{array}\right]$$

where *N *is the total number of states, subject to the normalisation condition $\underset{i}{\sum }\pi_{i}=1$. Then Eqn.12 becomes:

(15) $$\begin{array}{c} {\text{Π}}^{\prime }=\text{Π}*Q=\left[\begin{array}{cccc} \pi_{1} & \pi_{2} & \cdots & \pi_{N}\\ \pi_{1} & \pi_{2} & \cdots & \pi_{N}\\ \vdots & \vdots & & \vdots \\ \pi_{1} & \pi_{2} & \cdots & \pi_{N} \end{array}\right]\;\left[\begin{array}{cccc} q_{S_{1},S_{1}} & q_{S_{1},S_{2}} & \cdots & q_{S_{1},S_{N}}\\ q_{S_{2},S_{1}} & q_{S_{2},S_{2}} & \cdots & q_{S_{2},S_{N}}\\ \vdots & \vdots & & \vdots \\ q_{S_{N},S_{1}} & q_{S_{N},S_{2}} & \cdots & q_{S_{N},S_{N}} \end{array}\right]=0\\ \;\;\;\;\;\left[\begin{array}{cccc} \pi_{1} & \pi_{2} & \cdots & \pi_{N} \end{array}\right]\;\left[\begin{array}{c} q_{S_{1},S_{i}}\\ q_{S_{2},S_{i}}\\ \vdots \\ q_{S_{N},S_{i}} \end{array}\right]=0\;\text{ for }\;i=1,\ldots ,N \end{array}$$

As $q_{S_{i},S_{i}}=-\underset{j\neq i}{\sum }q_{S_{i},S_{j}}$, then Equ.15 can be simplified to:

(16) $$\underset{\text{flux}\;\text{out}\;\text{of}\;\text{state}\;\;S_{i}}{\underset{︸}{\pi_{i}*\underset{j\neq i}{\sum }q_{S_{i},S_{j}}}}=\underset{\text{flux}\;\text{into}\;\text{state}\;\;S_{\text{i}}}{\underset{︸}{\underset{j\neq i}{\sum }\pi_{j}*q_{S_{j},S_{i}}}}$$

We can therefore use Eqn.10,16 to obtain steady state distribution.

**Figure 3:**
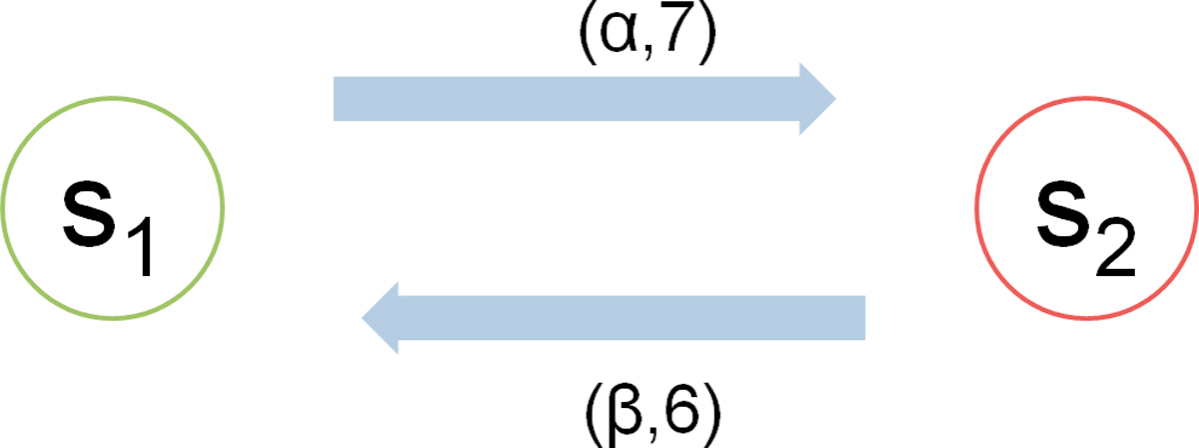
**The state transition diagram of a two state Markov process**.

Here is a simple example to explain the above process. Suppose the system behaviour can be modeled by a two state Markov process whose state transition diagram is shown in Figure 3. Then the generator matrix is as follows:

(17) $$Q=\left[\begin{array}{cc} -7 & 7\\ 6 & -6 \end{array}\right]$$

We can therefore get steady state distribution from:

(18) $$\begin{array}{c} \pi_{1}*7=\pi_{2}*6\\ \;\;\pi_{1}+\pi_{2}=1 \end{array}$$

which is:

(19) $$\pi =\left[\pi_{1},\;\pi_{2}\right]=\left[0.46,0.54\right]$$

#### Calculating the resource utilisation and system throughput

The steady state distribution *π_i _*stands for the proportion of time that the process spends in state *S_i_*. By finding in which state *S_i _*the resource is busy, we can calculate the resource utilisation. For example, if the resource at *place_i _*is busy in state *S*_1_, *S*_3 _and *S*_4_, then its utilisation can be obtained by *π*(*Resource_busy_placei_*) = *π*(1) + *π*(3) + *π*(4). If this resource has multiple copies, we can calculate its average utilisation by

(20) $$Utilisation_{Resource_{placei}}=\left(\overset{Num_{Resourcei}}{\underset{j=1}{\sum }}\pi {\left(Resource\text{\_}busy_{placei}\right)}_{j}\right)/Num_{Resource_{placei}}$$

where $Num_{Resource_{placei}}$ is the total number of *Resource_placei _*copies.

In the clinical pathway model, suppose that there is a patient who completes the activity with the action type λ leaves the hospital. The throughput of pathway is estimated by the expected number of completed activities with action type *λ*. Given the definition of resource *Resource_placeN _*in the last place of the care process as follows:

(21) $$\begin{array}{ccc} Resource_{-}idle_{placeN}\; & \overset{def}{=}\;\left(\sigma ,\;rate_{\sigma }\right).Resource_{-}busy_{placeN} & \\ Resource_{-}busy_{placeN}\; & \overset{def}{=}\;\left(\lambda ,\;rate_{\lambda }\right).Resource_{-}idle_{placeN} \end{array}$$

where the completion of activity (λ, *rate*_λ_) enables the resource to become idle. Then the throughput *T *can be represented by:

(22) $$T=rate_{\lambda }*Utilisation_{Resource\text{\_}busy_{placeN}}$$

showing that the system throughput is associated with the utilisation of the resource whose state transition depends on the type λ activity.

**Figure 4:**
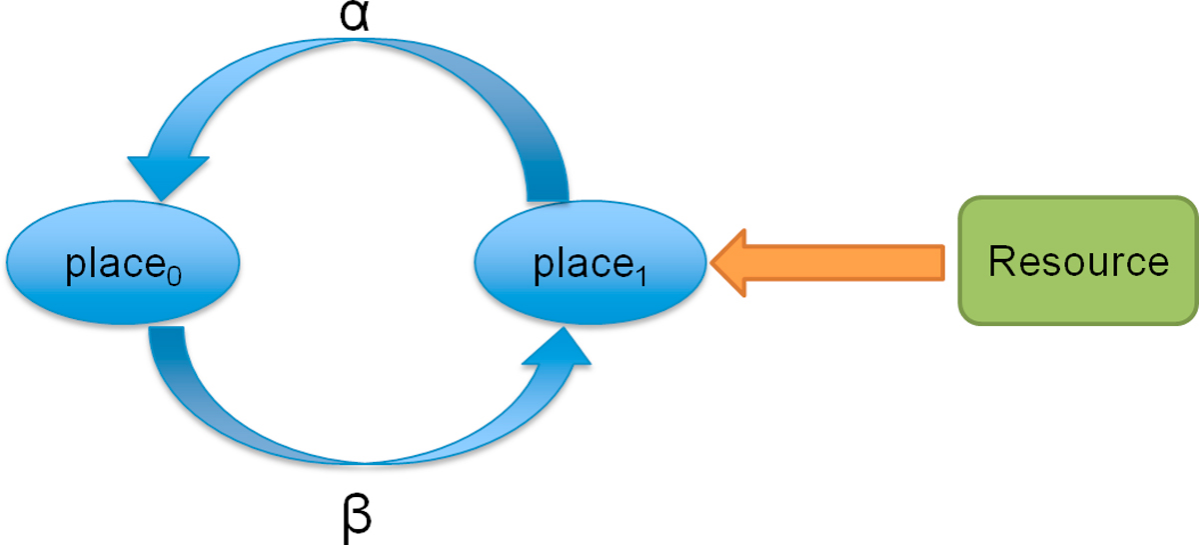
**A simple clinical pathway**. Patient in the *place*_0 _can be transferred to *place*_1 _with the completion of *β *activity. When the resource in the *place*_1 _is available, patient can be treated and returns to *place*_0 _after *α *activity.

**Figure 5:**
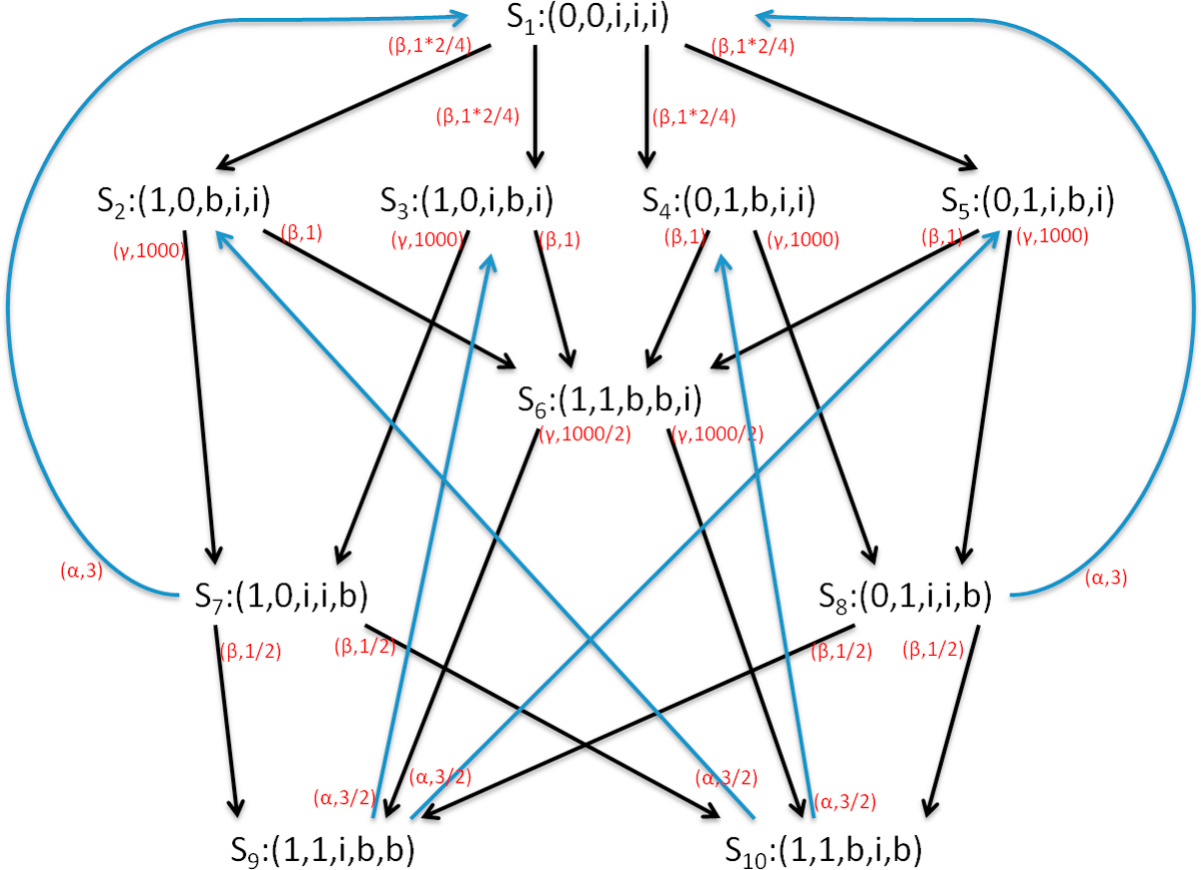
**The state transition diagram of a simple CPP model**.

Let us introduce the CPP model for a simple clinical pathway (see Figure 4) to show how to analyze the resource utilisation and throughput. Suppose the CPP model is defined as follows:

The equilibrium state can only be maintained if the Markov process is irreducible that every state can be reached from all other states. Therefore CPP constructs a cyclic model in which the discharged patient will return to its initial state *Patient*_*place*0_. The state space of this simple CPP model is shown in Figure 5, where each state is represented by a *five-tuple*. For example, in state *S*_5_: (0, 1, *i*, *b*, *i*), the first element 0 represents the first patient is in the *place*_0_; the second element 1 represents the second patient is in the *place*_1_; the third element *i *represents the first waiting room is idle; the fourth element *b *represents the second waiting room is busy; and the last element *i *represents the resource in the *place*_1 _is idle.

The generator matix *Q *has the following form:

(24) $$Q=\left[\begin{array}{cccccccccc} -2 & 0.5 & 0.5 & 0.5 & 0.5 & 0 & 0 & 0 & 0 & 0\\ 0 & -1001 & 0 & 0 & 0 & 1 & 1000 & 0 & 0 & 0\\ 0 & 0 & -1001 & 0 & 0 & 1 & 1000 & 0 & 0 & 0\\ 0 & 0 & 0 & -1001 & 0 & 1 & 0 & 1000 & 0 & 0\\ 0 & 0 & 0 & 0 & -1001 & 1 & 0 & 1000 & 0 & 0\\ 0 & 0 & 0 & 0 & 0 & -1000 & 0 & 0 & 500 & 500\\ 0 & 0 & 0 & 0 & 0 & 0 & -4 & 0 & 0.5 & 0.5\\ 3 & 0 & 0 & 0 & 0 & 0 & 0 & -4 & 0.5 & 0.5\\ 0 & 0 & 1.5 & 0 & 1.5 & 0 & 0 & 0 & -3 & 0\\ 0 & 1.5 & 0 & 1.5 & 0 & 0 & 0 & 0 & 0 & -3 \end{array}\right]$$

from which we can get the steady state distribution:

(25) $$\begin{array}{ccc} & \left[\pi_{1},\;\pi_{2},\;\pi_{3},\;\pi_{4},\;\pi_{5},\;\pi_{6},\;\pi_{7},\;\pi_{8},\;\pi_{9},\;\pi_{10}\right] & \\ & =\left[0.5284,\;3.523*10^{-4},3.523*10^{-4},3.523*10^{-4},3.523*10^{-4},1.409*10^{-6},\;0.1761,\;0.1761,\;0.05895,\;0.05895\right] \end{array}$$

Therefore, we can obtain resource utilisation and throughput:

(26) $$\begin{array}{ccc} Utilisation_{Resource_{place1}}\; & =\;\pi \left(Resource_{-}busy_{place1}\right)=\pi_{7}+\pi_{8}+\pi_{9}+\pi_{10}=0.47 & \\ T\; & =\;3*Utilisation_{Resource_{place1}}=1.41 \end{array}$$

We can use PEPA eclipse plugin [30] to simulate the developed CPP model, from which resource utilization and throughput can be directly obtained.

#### Critical assessments-state explosion problem

When there are many patients and multiple copies of resources involved in the system description part, the state explosion problem occurs. For instance, when there are eight patients, eight waiting rooms and three resources in Figure 4 clinical pathway, the number of states increases from 10 to 75582. Then the dimension of generator matrix *Q *becomes 75582 × 75582. Hence, calculating steady state distribution by solving Eqn.16 turns out to be computational intensive and requires large storage space. To address these problems, this paper uses two methods which are state aggregation and fluid analysis.

It has been pointed out in [31,32], by exploiting the strong equivalence relations, we can generate the aggregated CTMC where the number of states can be reduced. Consider again the simple CPP model in Eqn.23. As the states *S*_2_, *S*_3_, *S*_4 _and *S*_5 _shows that there is one patient in *place*1, one patient in *place*0, one waiting room is busy and the resource is idle, the aggregated state $S_{2}^{\prime }$ can be used to represent them without considering which patient is in *place*1 and which waiting room is occupied:(27)

Similarly, we use $S_{4}^{{}^{\prime }}$ to represent *S*_7 _and *S*_8_, and $S_{5}^{{}^{\prime }}$ to represent *S*_9 _and *S*_10_:(28)

**Figure 6:**
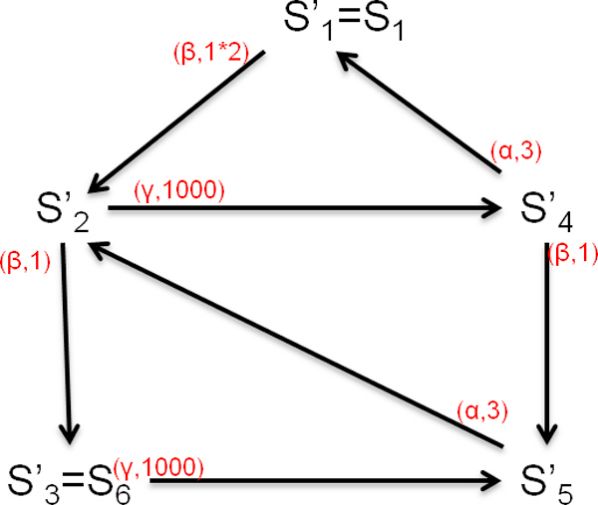
**The aggregated state transition diagram of a simple CPP model**.

Therefore, the number of aggregated states is reduced to 5. The state transition diagram is shown in Figure 6, and the generator matrix *Q*' is as follows:

(29) $$Q^{\prime }=\left[\begin{array}{ccccc} -2 & 2 & 0 & 0 & 0\\ 0 & -1001 & 1 & 1000 & 0\\ 0 & 0 & -1000 & 1000 & 0\\ 3 & 0 & 0 & -4 & 1\\ 0 & 3 & 0 & 0 & -3 \end{array}\right]$$

We can get steady state distribution as:

(30) $$\pi =\left[\pi_{1},\;\pi_{2},\;\pi_{3},\;\pi_{4},\;\pi_{5}\right]=\left[0.5284,0.001409,\;1.409*10^{-6},0.3523,0.1179\right]$$

and further obtain resource utilisation and throughput:

(31) $$\begin{array}{ccc} Utilisation_{Resource_{place1}} & =\pi \left(Resource\text{\_}busy_{place1}\right)=\pi_{4}+\pi_{5}=0.47 & \\ T & =3*Utilisation_{Resource_{place1}}=1.41 \end{array}$$

which are similar with Eqn.26.

Although the number of states can be reduced by using the aggregation method (implemented in PEPA eclipse plugin), when the amounts of patients and resources become extremely large, the state aggregation method cannot solve the state explosion problem well. Hence, fluid analysis of stochastic process model is used in [27] to analyze systems of size 10^1000 ^states and beyond. This approach approximates the state space by using a set of ordinary differential equations to describe the time evolution of state components. Assume that, the mean number of component *P_i _*at time *t *is denoted as *N *(*P_i_*(*t*)), then a differential equation can be used to represent the changes in *N*(*P_i_*(*t*)) as:

(32) $$N{\left(P_{i}\left(t\right)\right)}^{\prime }=-\underset{j:P_{i}\underset{\to }{(\alpha .,)}P_{j}}{\sum }\text{rate of }\alpha \text{ - action}\;\text{leaving }P_{i}+\underset{j:P_{j}\underset{\to }{(\beta .,)}P_{i}}{\sum }\text{rate of }\beta \text{ - action}\;\text{entering }P_{i}$$

A set of differential equations to present the CPP model in Eqn.23 is as follows:

(33) $$\begin{array}{ccc} N{\left(Patient_{place0}\left(t\right)\right)}^{\prime }\; & =\;-1*min\left(N\left(Patient_{place0}\left(t\right)\right),\;N\left(Wait\text{\_}room0\text{\_}idle\left(t\right)\right)\right) & \\ & \;+3*N\left(Resource\text{\_}busy_{place1}\left(t\right)\right)\\ N{\left(Patient_{place1}\left(t\right)\right)}^{\prime }\; & =\;1*min\left(N\left(Patient_{place0}\left(t\right)\right),\;N\left(Wait\text{\_}room0\text{\_}idle\left(t\right)\right)\right)\\ & \;-3*N\left(Resource\text{\_}busy_{place1}\left(t\right)\right)\\ N{\left(Wait\text{\_}room0\text{\_}idle\left(t\right)\right))}^{\prime } & =-1*min\left(N\left(Wait\text{\_}room0\text{\_}idle\left(t\right)\right)\right),N\left(Patient_{place0}\left(t\right)\right))\\ & \;+1000*N\left(Resource_{idle}\left(t\right)\right),\;N\left(\left(Wait\text{\_}room\text{\_}busy\left(t\right)\right)\right)\\ N{\left(Wait\text{\_}room0\text{\_}busy\left(t\right)\right))}^{\prime } & =1*min\left(N\left(Wait\text{\_}room0\text{\_}idle\left(t\right)\right)\right),N\left(Patient_{place0}\left(t\right)\right))\\ & \;-1000*N\left(Resource_{idle}\left(t\right)\right),\;N\left(\left(Wait\text{\_}room0\text{\_}busy\left(t\right)\right)\right)\\ N{\left(Resource\text{\_}idle_{place1}\left(t\right)\right)}^{\prime } & =3*N\left(Resource\text{\_}busy_{place1}\left(t\right)\right)\\ & \;-1000*min\left(N\left(Resource\text{\_}idle_{place1}\left(t\right)\right),\;N\left(Wait\text{\_}room0\text{\_}busy\left(t\right)\right)\right)\\ N{\left(Resource\text{\_}busy_{place1}\left(t\right)\right)}^{\prime } & =-3*N\left(Resource\text{\_}busy_{place1}\left(t\right)\right)\\ & \;+1000*min\left(N\left(Resource\text{\_}idle_{place1}\left(t\right)\right),\;N\left(Wait\text{\_}room0\text{\_}busy\left(t\right)\right)\right) \end{array}$$

where the initial conditions are:

(34) $$\begin{array}{cc} N\text{(}Patient_{place0}(0))\; & \text{ = }\;\text{2}\\ N\text{(}Patient_{place1}(0))\; & \text{ = }\;\text{0}\\ N(Wait_{-}room0_{-}idle\left(0\right)) & =2\\ N\;\text{(}Wait_{-}room{\text{0}}_{-}busy(0)) & =\text{0}\\ N\text{(}Resource_{-}idle_{place\text{1}}(0)) & =\text{1}\\ N\text{(}Resource_{-}busy_{place\text{1}}(0)) & =\text{0} \end{array}$$

**Figure 7:**
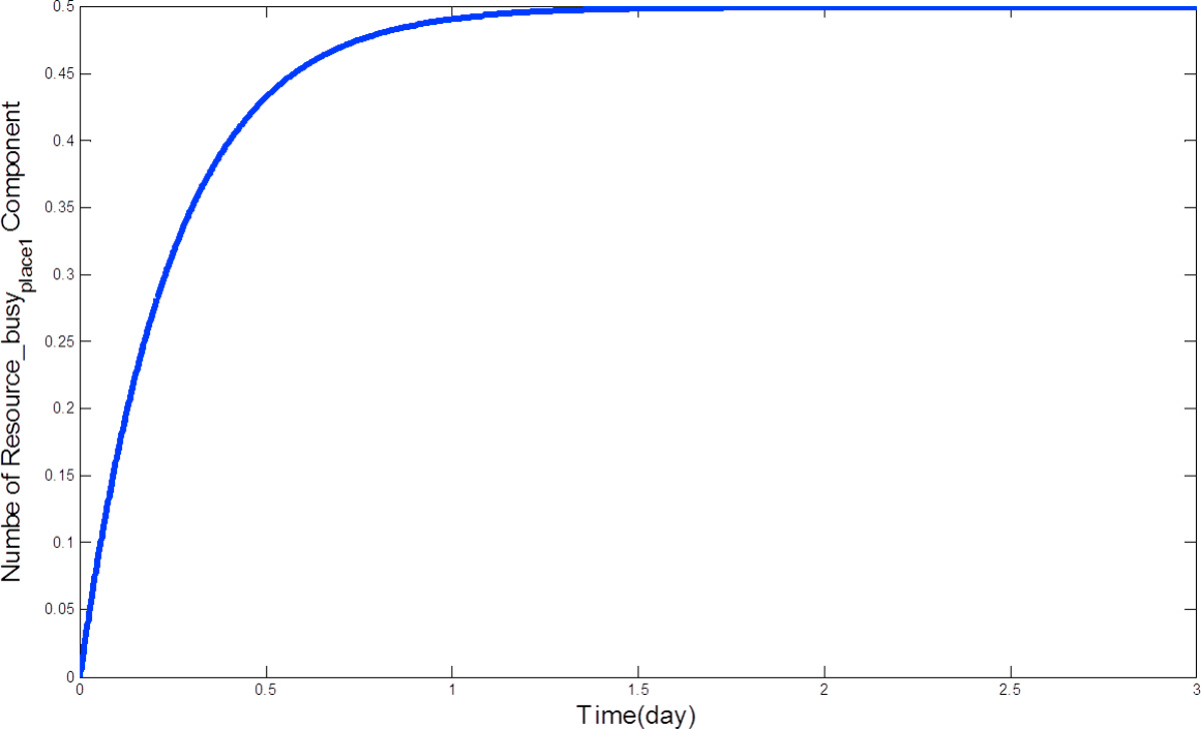
**The numerical solution to the ODEs representing the mean number of *Resource_busy*_*place*1 _over time**.

By solving these equations, we can obtain the time evolution of each component. The details of fluid analysis can be found in [33]. By exploring the time evolution of *P_i_*, we can find the mean time that *P_i _*reaches its maximum. For example, Figure 7 shows *N*(*Resource_busy*_*place*1_(*t*)), whose maximum can be reached within two days. With the introduction of fluid analysis, state explosion problem can be addressed.

#### Calculating passage time

Based on fluid analysis, the mean passage time of a patient to be discharged can be estimated by using a stochastic probe [34]. For example, if we are interested in the time by which a *Patient*_*place*0 _component has done its first *α *activity, we can attach a probe to *Patient*_*place*0 _to remember whether it has performed *α *activity. The probe can be defined as follows:

(35) $$\begin{array}{ccc} NotFinished\; & \overset{def}{=}\;\left(\alpha ,3\right).Finished & \\ Finished\; & \overset{def}{=}\;\left(\alpha ,3\right).Finished \end{array}$$

We can replace the *Patient*_*place*0 _component in the system description part of the CPP model by the synchronised component . The modified differential equations are:

where the initial conditions are:(37)

**Figure 8:**
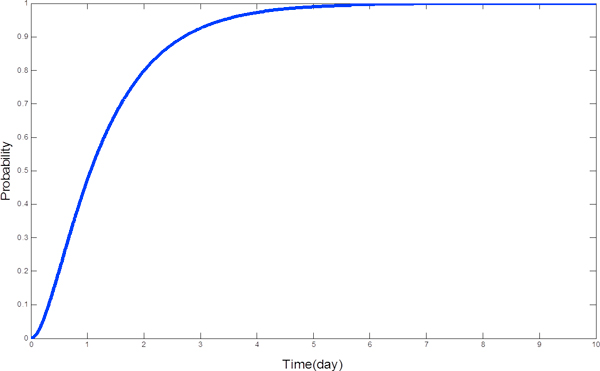
**The CDF of the passage time of a patient performing the first *α *action**.

By summing the counts of all patient components of the form , we can get an approximation to the cumulative density function (CDF) for the time it takes for an individual *Patient*_*place*0 _component to perform its first *α *action (see Figure 8). In this paper, we use a tool, *Grouped PEPA Analyser*, developed in [35] to simulate the CPP model. This tool can estimate resource utilisation and passage time based on fluid analysis.

## Results

In this section, the CPP modelling method is applied to model the stroke clinical pathway. It begins with the parameter settings of experiment. Then detailed description of the CPP model for stroke clinical pathway is shown. By simulating the pathway using the CPP modelling method, we can optimize the resource allocation, estimate the passage time and find the maximum throughput with the current resource distribution.

### Experiment setup

In order to improve stroke service in London, the NHS investigates seven HASUs opened in 2010 including the one in Charing Cross Hospital. To ensure appropriate and consistent measurements, the SINAP database is used. Among the datasets published by NHS [36], the HASU activity data which contains clinical information for HASU in Charing Cross Hospital is shown in Table 1. It can be used to calculate transition probabilities in Figure 1. For example, there are 116 stroke patients, 27 stroke mimic patients and 15 TIA patients. Then in Figure 1, the transition probability for the patient from A&E to A&E referral is 27/(116+15+27) = 17%. Similarly, the transition probability from CT scan to A&E referral is 15/(116+27) = 10%.

**Table 1:** HASU activity data from NHS [36]

Number of beds in HASU	Number of stroke admissions	Number of 'mimic' admissions	Number of Transient Ischaemic Attack (TIA)	Number of patient directly admitted to a HASU
20	116	27	15	156

**Number of stroke patient thrombolysed**	**Average door to needle (thombolysis) times (min)**	**Number of patients receiving a brain scan within 24hrs of admission to HASU**	**HASU median length of stay (day)**	**Total number discharged home directly from HASU**

11	58	158	2	48

To build the formal model of stroke clinical pathway, parameters involved in pathway including execution time of each care activity and the number of resources available at each department should be estimated in advance. Some of them can be directly obtained from Table 1, where we can find the number of beds available in HASU is 20 and the median length of stay in HASU is 2 days. Moreover, NHS records the average time from 999 call to arrive hospital to be 62 minutes. As the FAST test is carried out on ambulance, the mean time of FAST test is therefore assumed to be 1 hour. Other parameters such as the number of stroke teams in A&E and CT scanners are not extracted directly from NHS databases, and they are estimated by consulting related documents and stroke experts. In this paper, we assume that there are 3 stroke teams and 3 CT scanners in Charing Cross Hospital. The number of ambulances is set to be unlimited, ignoring the influence of the ambulance in the model. Moreover, the mean lengths of stay in A&E and CT scan departments are assumed to be 0.75 hour and 3 hours, respectively. All the parameters and their value are summarized in Table 2.

**Table 2:** The settings of experiment

Department	Resource	Resource Number	Activity	Activity Period	Rate (/(day*resource))
LAS	Ambulance	Unlimited	do Fast	1 hour	24

A&E for stroke	Stroke team	3	stroke investigation	0.75 hour	32

CT scan	Scanner	3	scan	3 hours	8

HASU	bed	20	rehabilitation	48 hours	0.5

**Figure 9:**
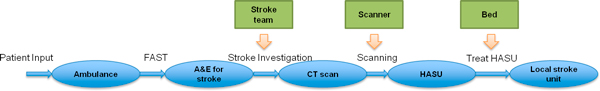
**Simplified stroke clinical pathway**.

This paper applies CPP to model the main stream of the pathway where only the patients who can finally go to the HASU are analyzed. Hence the pathway in Figure 1 is simplified as it is shown in Figure 9.

### The CPP model of the stroke clinical pathway

The pathway model contains three parts, which are state definition, resource specification and system description. The CPP representation of these three parts is as follows.

1. State definition

(38) $$\begin{array}{ccc} Patient_{Home}\; & \overset{def}{=}\;\left(have\text{\_}stroke,\;r_{income}\right)\;.Patient_{LAS}; & \\ \;\;\;Patient_{LAS}\; & \overset{def}{=}\;\left(do\text{\_}FAST,\;r_{do\text{\_}FAST}\right)\;.Patient_{A\text{\_}E\text{\_}Re\text{s}us};\\ Patient_{A\text{\_}E\text{\_}Re\text{s}us} & \overset{def}{=}\left(asses\text{\_}and\text{\_}investigate,r_{assess\text{\_}and\text{\_}investigate}\right).Patient_{CT\text{\_}scan};\\ \;\;\;Patient_{CT\text{\_}scan}\; & \overset{def}{=}\;\left(scan,\;r_{scan}\right)\;.Normal\text{\_}treat;\\ Patient_{Normal\text{\_}treat}\; & \overset{def}{=}\left(treat\text{\_}HASU,\;r_{treat\text{\_}HASU}\right)\;.Patient_{Home}; \end{array}$$

2. Resource specification

(39) $$\begin{array}{ccc} Stroke\text{\_}team\text{\_}idle\; & \overset{def}{=}\;\left(do\text{\_}FAST,\;r_{do\text{\_}FAST}\right).Stroke\text{\_}team\text{\_}busy; & \\ Stroke\text{\_}team\text{\_}busy\; & \overset{def}{=}\;\left(assess\text{\_}and\text{\_}investigate,\;r_{assess\text{\_}and\text{\_}investigate}\right).\;Stroke\text{\_}team\text{\_}idle;\\ Wait\text{\_}room1\text{\_}idle\; & \overset{def}{=}\;\left(assess\text{\_}and\text{\_}investigate,\;r_{assess\text{\_}and\text{\_}investigate}\right).Wait\text{\_}room1\text{\_}busy;\\ Wait\text{\_}room1\text{\_}busy\; & \overset{def}{=}\;\left(wait\text{\_}scan,\;r_{wait\text{\_}for\text{\_}calling}\right).Wait\text{\_}room1\text{\_}idle;\\ Scan\text{\_}idle\; & \overset{def}{=}\;\left(wait\text{\_}scan,\;r_{wait\text{\_}for\text{\_}calling}\right).\;Scan\text{\_}busy;\\ Scan\text{\_}busy\; & \overset{def}{=}\;\left(scan,\;r_{scan}\right)\;.Scan\text{\_}idle;\\ Wait_{-}room2_{-}idle\; & \overset{def}{=}\;\left(scan,\;r_{scan}\right)\;.Wait_{-}room2_{-}busy;\\ Wait_{-}room2_{-}busy\; & \overset{def}{=}\;\left(wait\text{\_}bed,\;r_{wait\text{\_}for\text{\_}calling}\right)\;.Wait_{-}room2_{-}idle;\\ Bed\text{\_}idle\; & \overset{def}{=}\;\left(wait\text{\_}bed,\;r_{wait\text{\_}for\text{\_}calling}\right).\;Bed\text{\_}busy;\\ Bed\text{\_}busy\; & \overset{def}{=}\;\left(treat\text{\_}HASU,\;r_{treat\text{\_}HASU}\right).\;Bed\text{\_}idle; \end{array}$$

3. System description The third part is the system description as follows:(40)

In order to get the average patient incoming rate to 4, the parameter *Num_patient _*is set to 1000 and *r_income _*is 0.004.

### Three performance analysis scenarios

Three scenarios are tested in this subsection to show the application of the CPP model in performance analysis. The CPP model can detect the optimal resource allocation, estimate the passage time and determine the maximum throughput of the healthcare system.

#### Scenario 1: Resource optimization

**Figure 10:**
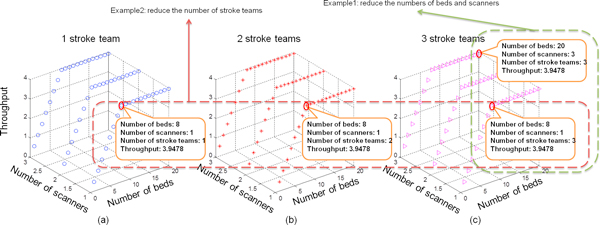
**Resource optimization of clinical pathway**. The number of scanners varies from 1 to 3 and the number of beds ranges between 1 and 20 in each graph. The number of stroke team is set to 1 in (a), 2 in (b) and 3 in (c) respectively. The maximum throughput of 3.95 can be achieved in (a), where there are 1 stroke team, 1 scanner and 8 beds.

Initially there are 3 stroke teams, 3 scanners and 20 beds available in the stroke clinical pathway. As the average patient input is around 4, it may happen that the resource utilisation is low. Hence, we investigate the influence of resource quantity on system throughput. By varying the numbers of stroke teams, scanners and beds, we can find from Figure 10 that the largest throughput that can be achieved is 3.95 *patients/day*, slightly smaller than 4 *patients/day*. We cannot have the maximum throughput value exactly equal to the number of input patients. It can be explained from the definition of parallel input patients *Patient_Home_*[*Num_patient_*], where *Num_patient _*is 1000 and the input rate is 0.004. Theoretically, the number of input patients per day should be 1000 * 0.004 = 4. On the first day, 4 patients on average fall ill and are treated sequentially through the clinical pathway. As the mean time for the patient staying in HASU department is 2 days, it is possible that these 4 patients are still in the pathway on the next day. Hence, they cannot return to the state *Patient_Home _*on the second day, meaning that the number of patients at *Patient_Home _*is slightly smaller than 1000. Therefore, there is a trivial difference between the maximum throughput and the number of input patients. However, this small difference does not influence the performance analysis process.

Figure 10 is used to show how our performance analysis result guides medical staff to find optimal resource allocation, i.e., the amount of resource is kept minimal while still maintaining the maximum throughput in the clinical pathway. The three 3D graphs look similar and they actually demonstrate the process of reducing redundant resources while keeping the throughput unchanged. In these 3D graphs, we give two examples. In the first example, the numbers of beds and scanners are reduced from 20/3 to 8/1, respectively. This example shows that twelve redundant bed resources and two scanner resources can be removed and the clinical pathway's throughput is not influenced. The second example further reduces two redundant stroke team resources and keeps the throughput unchanged.

We can obtain the optimal number of each resource in Table 3 and find that the resource utilisation can be increased while the system throughput is maintained. It is notable that the CPP model can find the optimal resource allocation with varying number of input patients. For example, with an input of 4 patients per day, the optimal resource allocation is 1 stroke team, 1 scanner and 8 beds; if the input rate increases to 0.006, meaning that there are 6 patients falling ill every day, the optimal resource allocation is found to be 1 stroke team, 1 scanner and 12 beds.

**Table 3:** Comparison of resource utilisation: The utilisations of resources resulting from the model with initial and optimal parameter sets are compared.

	Stroke team		Scanner		Bed	
	
	Number	Utilisation	Number	Utilisation	Number	Utilisation
Initial parameter set	3	0.038	3	0.153	20	0.368

Optimal parameter set	1	0.115	1	0.46	8	0.92

#### Scenario 2: Passage time estimation

Besides estimating the system throughput and resource utilisation, CPP model can further predict the mean passage time of a patient with the introduction of a stochastic probe as follows:

(41) $$\begin{array}{ccc} NotFinished\; & \overset{def}{=}\;\left(treat\text{\_}HASU,\;r_{treat\text{\_}HASU}\right).\;Finished; & \\ Finished\; & \overset{def}{=}\;\left(treat\text{\_}HASU,\;r_{treat\text{\_}HASU}\right).\;Finished; \end{array}$$

**Figure 11:**
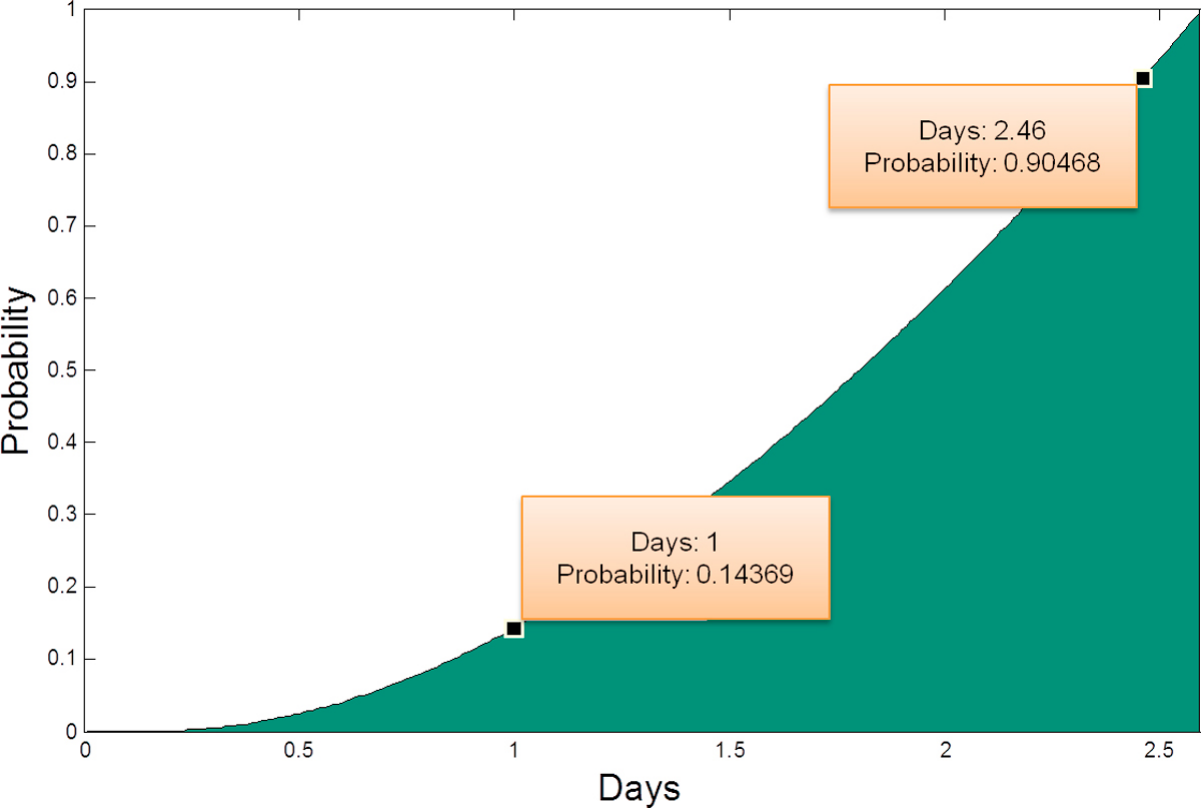
**Passage time**. It shows the probability that a patient can be discharged within a specific time. This figure is obtained when the model uses an optimal parameter set. When the model uses the initial parameter set, we get the same figure, showing that the system passage time remains the same.

The state *NotFinished *becomes *Finished *once the activity whose action type is *treat_HASU *is completed. When we attach this probe to the patient state component as follows:(42)

the mean time of a patient from falling ill to leaving the HASU can be obtained. Figure 11 shows the probability that a patient is discharged from the HASU within a specific time duration when there is 1 stroke team, 1 scanner and 8 beds available. We can see that 90% of patients can be discharged within 2.5 days and only 14% of patients can be discharged within 1 day. Estimation of the whole passage time not only tells patients when they can be discharged, but also informs doctors the resource occupation time.

#### Scenario 3: Maximum input estimation

**Figure 12:**
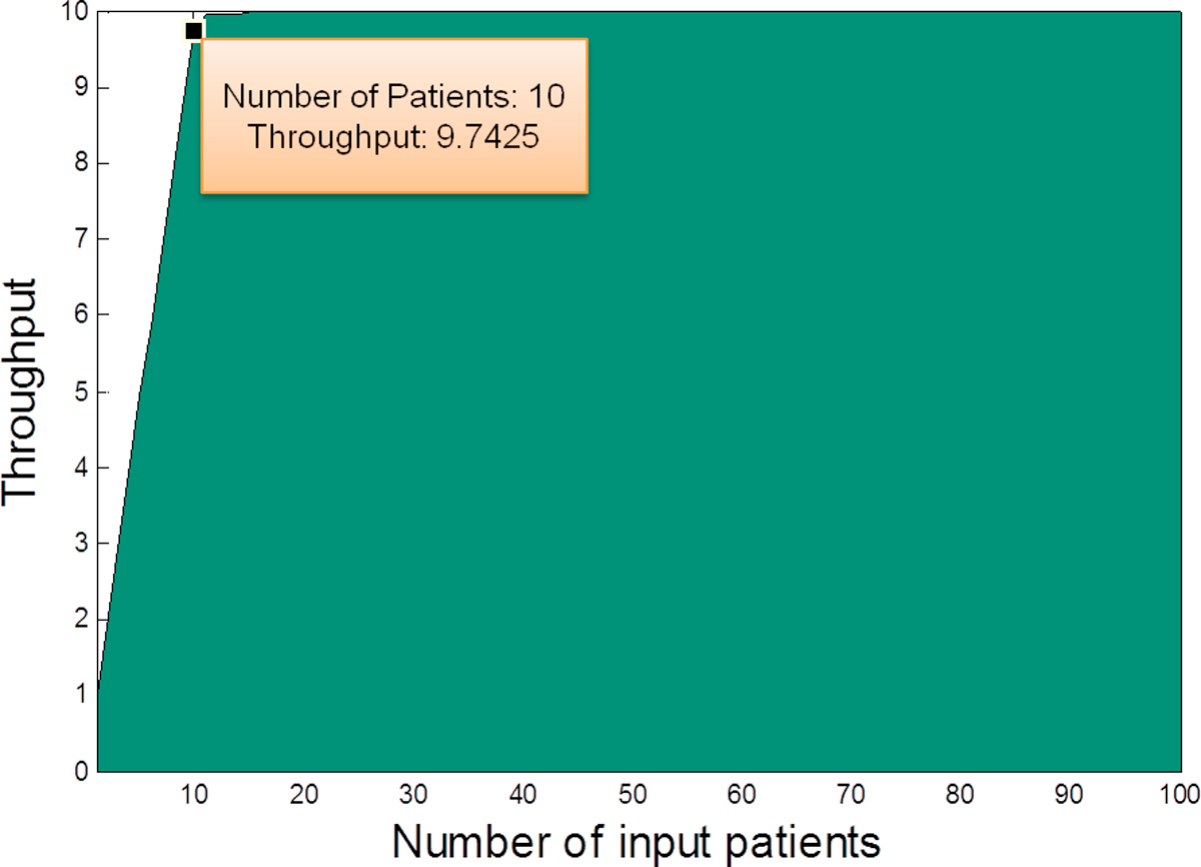
**Maximum throughput**. By varying the number of input patients, the maximum system throughput can be obtained, where the model uses the initial parameter settings.

We can also examine the maximum throughput with different parameter settings. For example, if the pathway model uses the initial parameters, in Figure 12 we can see that when the number of input patients increases from 1 to 100, the maximum throughput is around 10 *patients/day*. When the input number is 10, the throughput reaches 9.74 *patients/day*, slightly smaller than the maximum value (this difference has been discussed in the *Resource optimization *subsection). Any further increase in the number of input patients has trivial contribution to the system throughput.

Therefore, by simulating the CPP model, we can find the maximum number of input patients that can be supported by the healthcare system. This maximum input estimation is significant for hospital to determine whether it can accept more stroke patients or not. For example, suppose there are already 10 patients on average coming to the hospital from the surrounding area. If the national health community asks whether this hospital can serve patients from larger area, meaning that more than 10 patients will arrive every day, by estimating the maximum input this hospital can determine whether this is possible and whether more resources are required to support the increased number of patients.

## Conclusions

This paper introduces a clinical pathway management platform, ICPA, whose core element is the stochastic model CPP. CPP can unambiguously describe a variety of elements in a clinical pathway. Using CPP, the clinical pathway can be quantitatively analyzed and this performance analysis can provide a range of useful information for facilitating clinical pathway management. A real-world stroke clinical pathway, obtained from Charing Cross hospital of Imperial College London, is employed to demonstrate the practical applicability of ICPA. Three scenarios were tested to show that ICPA can assist hospitals to improve healthcare system by 1) reducing redundant resources 2) predicting patient passage time 3) estimating the maximum patient input. The approach presented in this paper can be used effectively to manage the clinical pathways within one hospital. There are multiple revenues for extending the work and we discuss four possible directions below.

1. CPP model can be extended to incorporate survival analysis by collecting clinical data including patient recovery speed and survival rate. The augmented model can then be used to examine the influence of treatment delay on patients' recovery processes. Therefore, the results from the ICPA can be used to increase patient recovery probability and decrease the recovery time.

2. Multiple hospitals can be managed concurrently using ICPA. At present, there are seven hospitals in London with HASU departments to treat stroke patients. ICPA can be extended to combine their clinical data to build a uniform model. By analysing this model, patients can be dynamically scheduled to different hospitals in order to optimise their treatment process.

3. The stroke clinical pathway discussed in this paper is described in the coarse granularity. With clinical data, ICPA can be applied to analyse an element in the clinical pathway in the fine granularity. For example, the HASU department, one element of the stroke clinical pathway, needs to treat patients with multiple therapies. If this type of information can be obtained and modelled by CPP, ICPA can view HASU as a clinical pathway and conducts performance analysis on it.

4. A user friendly interface can be build to facilitate medical staff's access to ICPA. Currently, ICPA only provides analysis results such as optimal resource allocation to medical staff. By interpreting these performance analysis results, medical staff can optimally re-allocate medical resources and re-configure treatment process. In the future, we plan to develop a user portal to help medical staff construct the clinical pathway model by themselves.

## Competing interests

The authors declare that they have no competing interests.

## Authors' contributions

XY designed the method, ran simulation and drafted the manuscript. RH participated in the method design, analyzed the results and drafted the manuscript. YG defined the research theme and participated in the method design. JB performed simulation and drafted the manuscript. BC defined the research theme and helped to draft the manuscript. RD participated in the design and coordination. RK defined the research theme and participated in method design. All authors read and approved the final manuscript.
